# Emotional Prosody Effects on Verbal Memory in Euthymic Patients With Bipolar Disorder

**DOI:** 10.3389/fpsyt.2019.00466

**Published:** 2019-07-04

**Authors:** Mario Altamura, Licia Santamaria, Antonella Elia, Eleonora Angelini, Flavia A. Padalino, Claudia Altamura, Caterina Padulo, Nicola Mammarella, Antonello Bellomo, Beth Fairfield

**Affiliations:** ^1^Department of Clinical and Experimental Medicine, Psychiatry Unit, University of Foggia, Foggia, Italy; ^2^Department of Psychological, Health and Territorial Sciences, University of Chieti, Chieti, Italy

**Keywords:** bipolar disorder, vocal prosody, emotion, memory, recognition, comprehension

## Abstract

A growing body of evidence suggests that emotional prosody influences the ability to remember verbal information. Although bipolar disorder (BD) has been shown to be associated with deficits in verbal memory and emotional processing, the relation between these processes in this population remains unclear. In the present study, we aimed to investigate the impact of emotional prosody on verbal memory in euthymic BD patients compared with controls. Participants were randomly divided into three subgroups according to different prosody listening conditions (a story read with a positive, negative, or neutral prosody) and effects on a yes–no recognition memory task were investigated. Results showed that euthymic bipolar patients remembered comparable numbers of words after listening to the story with a negative or neutral prosody but remembered fewer words after listening to the positive version compared with healthy controls. Results suggest that verbal memory is hindered in BD patients after listening to the story read with a positive prosody. This recognition bias for information with a positive prosody may lead to negative intrusive verbal memories and poor emotion regulation.

## Introduction

The affective connotation attributed to a word or a sentence during speech includes a series of non-lexical cues, such as intonation, intensity, and timing that together or alone communicate the emotional reaction or state of the speaker (1, 2). This affective connotation, commonly called prosody, is crucial in communication since a word (or a sentence) can assume a positive or negative meaning independent of the valence of the words themselves. Indeed, prosody is a powerful contextual modulator of verbal content and emphasizes the importance of investigating how different populations of individuals process prosody.

In general, the ability to correctly identify, interpret, and use affective prosody emerges around the age of 9 years (3, 4) and then declines with age (5, 6). Older adults, in fact, generally have difficulty processing prosody [for a review, see Ref. (7)], although the reasons behind this difficulty remain controversial. Changes in bottom-up auditory processing may affect processing of prosody and, certainly, modifications in auditory perception often accompany the developmental trajectory (e.g., hearing loss, different voice pitch, pausing, and duration thresholds and discrimination). More interesting, differences in vocal emotional processes may also specifically affect the processing affective prosody beyond these auditory changes independent of age.

It is now widely recognized that affective information can have both enhancing and impairing effects on various cognitive processes (e.g., memory), depending on a range of factors. For instance, attentional and working memory capacity may play an important role in determining whether and how emotions influence declarative memory [see for reviews Refs. (8, 9)]. On the one hand, a large body of evidence suggests that affective information is generally processed and remembered better than neutral information (e.g., 10). For example, McGaugh and colleagues (11) demonstrated that stories with emotional content are remembered better than similar stories lacking emotional implications. On the other hand, affective content represents additional information that needs to be manipulated and may undermine rather than facilitate memory performance (12–14). Indeed, studies have suggested that focusing on affective prosody does not always translate into enhanced memory formation (15, 16) since the affective information contained in prosody may also be considered as an additional piece of information that needs to be processed, creating additional requests for cognitive resources (17). For example, Fairfield et al. (16) found that listening to a story read with an affective prosody hindered performance on a subsequent recognition task in healthy older adults compared with younger adults.

Last, some studies have found gender effects on emotion recognition showing that females outperformed males (18) (19). Indeed, it may be that females are generally the primary caretakers and may have evolved greater facility in identifying emotion due to their importance in long-term parental bonding (20). Other studies, however, failed to reproduce gender differences in emotion recognition ability (21, 22). In particular, a study by Ross and Monnot (23) found no gender differences in affective prosody recognition.

Regarding patients with bipolar disorders (BDs), numerous studies have found general impairments in working memory, and verbal and episodic memory in euthymic patients compared to healthy controls [for reviews, see Refs. (24–26)], whereas studies focusing on alterations in the perception of affective stimuli (27, 28) and the impact of affective information on memory in these individuals have produced divergent findings (29). For example, Kauer-Sant’Anna et al. (30) tested memory for affective and neutral story content in euthymic BD participants and healthy controls and found no memory enhancement for the affective content of the story compared with healthy controls. Differently, other studies found typical emotional enhancement effects in both remitted patients and healthy controls in facial recognition tasks and affective information processing (31–34). Thus, the exact nature and the role of affective information on memory in euthymic BD participants remain somewhat unclear.

More importantly, growing evidence suggests that BD patients are impaired in their ability to identify the affective prosody of neutral content sentences (35, 36). Some studies have found general deficits across emotion categories in both male and female populations (37, 38) and in remitted patients with BD (37, 39). Other studies have found more gender-specific deficits. Bozikas et al. (40) found impaired perception of affective prosody specific to females and to certain emotions (i.e., fear and surprise), whereas Van Rheenen and Rossell (36) found impaired emotional prosody recognition specific to men and for the processing of positive intonation. These deficits may be linked to deficits in emotion processing brain regions and a functional magnetic resonance imaging (fMRI) study suggested that patients with BD may lack the typical right lateralization temporal lobe response to emotional prosody compared to controls (41). Indeed, converging evidence indicates that the right hemisphere has a predominant role in processing emotions from the tone of voice (42, 43) and functional imaging studies have found the right prefrontal cortex plays a critical role in perceiving and recognizing emotional prosody (44, 45).

In sum, although some findings suggest that individuals with BD may have diminished perception of emotional prosody, no study to our knowledge has examined the effect of affective prosody on memory. In this study, we aimed to investigate emotional prosody effects on information processing and verbal memory in healthy adults and BD type I patients using a carefully controlled auditory procedure. In particular, since we were specifically interested in investigating how affective prosody is used during memory processing and remembering, we asked participants to listen to a brief story that contained neutral target words but that were read with a specific affective prosody (positive, negative, neutral) and then to recognize target words. In this manner, the task allowed us to investigate how listeners process affective prosody and to detect subsequent differences in the ability to discriminate words belonging to the story. We adopted a single story with a controlled neutral semantic content to avoid content-prosody interactions within the three versions. In this way, any differences could be attributed to the emotional connotation of the voice rather than the content of the story *per se* or, in other words, to the way the voice conveyed an emotional meaning to the text. This may help clarifying whether healthy adults and BD patients have specific preferences for affective information in auditory processing and whether this affects later processing. In addition, we used a between-subject design to avoid potential carryover effects of different vocal emotions on later processing. We predicted that although studies have shown that BD patients may have general working memory difficulties, in our study they may not show lower accuracy scores for general word memory since the recognition memory test was administered immediately after the study session. Instead, we expected difficulties with affective prosody in patients compared to healthy controls, but no group differences for word memory with neutral prosody in line with previous studies showing impaired memory retrieval for affective stimuli (30, 41, 46).

Understanding the effects of prosody on declarative memory in BD may provide critical insights into identifying potential factors that cause patients with BD to exhibit poor emotion regulation, to be vulnerable to relapse or to engage in risk taking behavior.

## Methods

### Participants

Participants included 48 individuals diagnosed with BD type I and a control group of 48 adults recruited from the local community, who did not meet current or past criteria for any Axis I disorder as defined by the *Diagnostic Manual of Mental Disorders* (DSM-5). BD diagnoses were confirmed using the structured clinical interview for DSM-5 (SCID-5-CV) by licensed clinical psychologists (47). Exclusion criteria for the bipolar patients included other axis I diagnoses. Exclusion criteria for all participants included report of a history of severe head trauma, stroke, neurological disease, severe medical illness, or alcohol or substance abuse in the past 6 months. Current symptoms of mania were measured using the Young Mania Rating Scale (YMRS) (48). Current symptoms of depression were measured using the Hamilton Depression Rating Scale (HAM-D) (49). To ensure symptom recovery, BD patients were included only if they had a score of ≤ 8 on the Hamilton Depression Rating Scale (HAM-D) and the Young Mania Rating Scale (YMRS) (40). The Positive and Negative Affect Schedule (PANAS) (50) was used to measure positive and negative state affect (current mood). The PANAS is a self-report tool commonly used to assess positive affect (PA) and negative affect (NA). The PANAS has two 10-item scales: one for PA and one for NA. Responses are rated on a 5-point scale from very slightly (1) to very much (5) (50). All participants completed the forward and backward digit spans of the Wechsler Adult Intelligence Scale-Revised measuring working memory capacity (WAIS-R; 51). Auditory acuity was evaluated using a screening procedure developed by Reilly et al. (52). To pass screening, participants had to successfully identify tones presented monaurally to either ear at 25 dB HL (1000, 2000, and 4000 Hz), followed by presentation of the same frequency tones to the contralateral ear. Controls and BD type 1 patients were randomly divided in three groups of 16 subjects each and assigned to one of the three prosody conditions (positive, negative and neutral). There were no differences between groups for general working memory abilities (controls: forward, *p* values > .12; backward digit, *p* values > .13; patients: forward, *p* values > .07; backward digit, *p* values > .09) nor for current mood (controls: positive PANAS, *p* values > .26; negative PANAS, *p* values > .55; patients: positive PANAS, *p* values > .36; negative PANAS, *p* values > .11). All participants took part in the study on a strictly volunteer basis and received no payment per participation. They received no other compensation. Healthy controls were recruited from the local Chieti area through a list of volunteers willing to participate in experiments in our lab. Patients were recruited from follow-up visits to a psychiatric unit at the University of Foggia.

Next, we conducted a *post hoc* power analysis with the program G*Power3 (53) to find out whether our experimental design had enough power to detect an effect of prosody on declarative memory in BD. The sample size of 96 was used for the statistical power analyses and alpha level of was *p* < .05. A *post hoc* power analysis revealed that on the basis of main effects, interaction and between-groups comparison the observed power was close to.86 with an observed effect size of *f* = .34 [medium-sized effect according to Cohen, (54)]. Approximately, 65 participants per group would be needed to obtain statistical power at the recommended .95 level and a large effect size [ *f* = .40; Cohen, (55)].

Participants gave written informed consent after the procedures were fully explained. Participants were not explicitly informed about the purpose of the study. The present study is in accordance with the Helsinki declaration and was approved by the local institutional review board.

### Materials and Procedures

We used a single neutral content short story composed of 112 words, 31 of which were neutral target words as previously done by Fairfield et al. (16). Target words were selected from the Italian version of the Affective Norms for Emotional Words (ANEW) (56) and had the following characteristics: mean frequency of use, 127.4 (SD = 97.9), mean valence of 5.07 (SD = 0.28) and mean arousal 5.03 (SD = 0.45). The story was read and recorded by a 28-year-old European professional actor in three versions that differed for affective prosody. The actor read one version with a happy prosody, one with a sad prosody, and the last with a neutral prosody. The positive version of the story lasted 72 s, the negative version lasted 72 s, and the neutral version lasted 70 s. Participants listened to the audio traces through a pair of Bose headphones in a room with a noise level below 25 dB. The three traces were presented at an average of 70 dB, comparable to the level of conversational speech. Pitch levels were within a range of 75 to 385 Hz. To evaluate the prosody manipulation, we carried out a preliminary rating study in our laboratory. An independent group of 30 older adults (M age = 70.9 years; SD = 4.9 years; 15 males) rated the three versions according to valence on a 7-point scale (1, absolutely negative to 7, absolutely positive). We chose to control the prosody manipulation in a group of older adults since much research has shown that emotion and affective information processing impairments emerge with age (57). Our reasoning was that if healthy older adults could correctly detect the prosody of each story, then the versions could be considered positive, negative, or neutral.

Prosody was effective in changing the direction of rating as participants rated the story as more positive (M = 6.4, SD = .68) when the actor read the story with a positive intonation, more negative (M = 2.1, SD = .74) with a negative intonation, and neutral (M = 4.2, SD = .75) when the story was read with no particular intonation, *F*_(2, 27)_ = 91,752, *p* < .001, η² = .87. Finally, to control for comprehension and intelligibility, we asked the independent group to answer five comprehension questions regarding the story. We did not find any difference in comprehension between prosody conditions (*F*_(2, 27)_ = .34, *p* = .71, *η*² = .02).

#### Study Phase

We adopted the same procedure as that used in a previous study (16). Participants listened to a short story and were instructed to pay attention to what the story was about. We presented our study as a comprehension study so participants did not know that a recognition memory test would follow.

#### Test Phase

The recognition phase began after a 3-min interval during which participants completed the Pattern Comparison Test (58). Participants were presented with a surprise yes-no auditory recognition memory task. For the recognition memory test, the 31 studied words were mixed with 20 new words to create a list of 51 items. The new words were comparable to the studied words, mean frequency of use, 121.9 (SD = 102.9), mean valence of 5.18 (SD = 0.26), and mean arousal 5.03 (SD = 0.60). To make the recognition phase comparable across groups, participants listened to the list of words read by an experimenter blind to the experimental hypothesis and who was not involved in recording of words. Participants answered “yes” if they thought they had heard the word during the story or “no” if they felt it was new. Before leaving, participants also answered five comprehension questions as an index of their ability to pay attention to the story.

## Results

Demographic and clinical characteristics of participants are presented in **Table 1**. All patients were receiving appropriate medication, lithium, atypical antipsychotics, and antidepressants, as monotherapy or in various combinations: 18 were on an atypical antipsychotic, 3 on classical antipsychotics, 40 on a mood stabilizer, and 3 on an antidepressant. Daily dosage of each antipsychotic was converted into a daily equivalent dosage of chlorpromazine (59). If patients were treated with a combination of antipsychotics, all obtained equivalent dosages of chlorpromazine were summed. All participants reported normal or corrected-to-normal visual and auditory acuity. We carried out a preliminary analysis for gender differences and comprehension scores. We did not find any significant effects regarding gender (*F*_(1,84)_ = .269, *p* = .61), so we excluded this variable from the analysis. We also collected accuracy to the five comprehension questions as an index of participants’ processing of the story. Accuracy in all participants was high > 60%.

**Table 1:** Demographic and clinical participant characteristics.

	BD (n = 48)	Controls (n = 48)	*P* values	α of Cronbach
Age (years)	46.7(11.6)	50.3 (5.9)	0.23	
Gender (male/female)	28/20	25/23	0.44	
Education	12.0 (2.1)	13.9 (2.9)	0.31	
Illness duration (years)	11.2 (8.6)			
Remission duration (months)	11.3 (15.2)			
YMRS	1.24 (1.6)			0.75
HAM-D	2.12 (3.2)			0.74
PANAS (positive affect)	30.5 (7.9)	30.6 (5.0)	0.43	0.87
PANAS (negative affect)	19.9 (7.8)	20.3 (7.4)	0.33	0.87
Forward digit span	5.8 (1.7)	6.7 (1.7)	0.85	
Backward digit span	4.6 (1.2)	5.8 (2.5)	0.02	
Chlorpromazine equivalent (mg/day)	146.62 (82.99)			

Young Mania Rating Scale (YMRS) (48).

Hamilton Depression Rating Scale (HAM-D) (49).

Positive and Negative Affect Scale (PANAS) (50).

We carried out a 2 (group: controls vs BD type 1 patients) × 3 (prosody: positive, negative, neutral) mixed ANOVA on recognition scores, calculated as hits-false alarms (HITs-FAs), that is the difference between the proportion of items correctly remembered and the proportion of new words recognized as old in line with the Signal Detection Theory (60). We found no main effect of group *F*_(1, 90)_ = 1,410, *p* = .24, *η*² = .02. Healthy controls and BD type 1 patients remembered a comparable number of words across all three conditions (positive, .33 vs. 26, p = .11; negative, .31 vs. 38, *p* = .20; neutral, .49 vs. 38, *p* = .09). There was a significant main effect of Prosody, F_(2, 90)_ = 5,805, *p* < .01, η² = .10. Accuracy improved when the story was read with a neutral prosody compared to the emotional prosody (.44 vs. .32). Finally, the two-way interaction between group and affect neared significance, *F*_(2, 90)_ = 3.063, *p* = .05; η² = .06. Planned comparisons revealed that both positive and negative prosody hindered recognition memory in healthy controls, whereas recognition memory was hindered in BD type 1 patients only after listening to the story read with a positive prosody (*p* < .001). Recognition accuracy is reported in **Table 2**.

**Table 2:** Recognition accuracy.

Group	Positive, M (SD)	Negative, M (SD)	Neutral, M (SD)
Healthy controls	.33 (.12)	.31 (.20)	.49 (.12)*^†^
BD patients	.25 (.16)**^†^	.38 (.13)	.38 (.24)

*Mean proportions and SD of correct recognition for group and affective prosody.*

*^†^neutral vs negative and positive p < 0.05.

**^†^p < 0.01 positive vs negative and neutral.

We used Pearson’s correlations to investigate the association between working memory capacity (as measured by forward and backward digit span) and performance on recognition task within the patient group only. There were no significant correlations between recognition accuracy and the two working memory tasks (*p* > 0.05 in all cases). Moreover, there were no significant correlations between recognition scores and chlorpromazine dosage equivalent (*p* > 0.05).

## Discussion

In this study, we examined the effects of the affective cues contained in prosody on verbal information processing and memory in BD patients and healthy controls with an affective prosody manipulation. To our knowledge, this is the first study to present detailed data on affective prosody effects on verbal memory among individuals with BD. The main results can be summarized as follows. First, both healthy participants and patients with BD remembered target words better when they were not embedded in an emotional vocal context (i.e., neutral vocal prosody). Second, the valence of affective prosody affected the pattern of recognition memory in patients and controls differently. Recognition performance was specifically affected by positive prosody in the BD patients who remembered fewer words after listening to the story read with a positive prosody respect to the patients who listened to the story on a negative or neutral prosody. Instead, healthy controls remembered fewer target words after listening to the story with a negative or a positive prosody.

Results showed a clear memory advantage for neutral prosody compared to affective prosody and are inconsistent with previous literature that shows how affective information is generally processed and remembered better than neutral information (61–64). Our divergent results might be due to differences in study stimuli and/or the nature of memory tested. Indeed, previous studies have generally focused on verbal memory impairments for words with positive or negative valence, whereas our study tested the effects of vocal prosody on neutral word recognition. Noteworthy, our findings are in line with previous prosody studies that revealed poorer memory for sentences spoken with a positive or negative intonation compared with a neutral one (65, 66). The deleterious effects of affective prosody on word recognition accuracy are consistent with the assumption that focusing on affective cues contained in prosody does not always guarantee better memory. In particular, it has been suggested that the vocal prosody might draw attention to non-verbal aspects of the stimulus (e.g., voice identity), thus leaving fewer resources for the encoding and consolidation of verbal aspects [e.g., Refs. (15, 16)]. Our findings suggest that these general effects of vocal prosody on word recognition may be relatively intact in both healthy adults and patients with BD.

The pattern of recognition as a function of the emotional prosody valence differed between patients and controls. Importantly, the differential responses between healthy subjects and euthymic bipolar patients cannot be explained by cognitive impairment in the patient group since we found no significant correlations between working memory performance and recognition scores. Furthermore, results cannot be explained by drug treatment since memory performance of patients was not significantly correlated. Recognition performance was specifically affected by positive prosody in the BD patients who remembered comparable numbers of words after listening to the story with a negative or neutral prosody but remembered fewer words after listening to the positive version. This diverges from previous emotional memory studies showing that participants with BD have impaired memory retrieval for negative emotional information (30, 46). However, it should be noted that those early studies examined the impact of affective stories on recall instead of affective cues contained in prosody. Our findings are in line with results obtained by Van Rheenen and Rossell (36) who demonstrated impaired ability to recognize positive intonations in patients with BD compared with healthy controls.

With regard to the nature of memory tested, results are in line with the transfer-appropriate processing theory that posits a general memory advantage when study and test processes match (67–69). In fact, our findings suggest that when participants listened to the story presented with a neutral prosody and were later tested for a series of old and new neutral items read with a neutral intonation, memory performance benefited from the neutral match between prosody and the words to be remembered. Differently, when prosody and words to-be-remembered were unmatched (affective prosody and neutral words), subsequent recognition memory was affected by the study phase intonation mismatch.

Regarding brain lateralization, another possible interpretation of our findings is that, contrary to healthy individuals, euthymic bipolar patients are constrained in their ability to engage affective processing of positive prosody, which may be related to a dysfunctional prefrontal regulation of emotional memory processing (41).

Finally, our study has several limitations. Our study includes prosody effects on only one neutral story and in a small sample. In particular, the small sample size was due to a limited number of patients who met inclusion criteria from the Psychiatry Unit in Foggia. Foggia is a small city in the southern part of Italy and accordingly receives a smaller number of psychiatric patients, in general, and BD patients, in particular. In line with the small number of eligible BD patients, we recruited only 48 healthy controls. Consequently, caution is recommended before placing too great a significance on the results without also considering effect sizes. Future studies should increase the number of participants and adopt a variety of stories to better specify prosody effects. Results of our study may reflect the different emotional intensity attributed to the processed intonations. Unfortunately, we did not ask the participants to rate the intensity of the material. Future experiments might request participants to rate the stimuli for both valence as well as for arousal. Despite these caveats, the present study provides important insights into underlying cognitive mechanisms associated with emotional prosody in BD. Specifically, results indicated that verbal memory was hindered in BD patients after listening to the story read with a positive prosody. The effects of emotional prosody on verbal memory exhibited by euthymic patients with BD are likely to have important clinical implications since identifying potential factors that cause patients with BD to exhibit poor emotion regulation may clarify vulnerability to relapse or risk-taking behavior. Moreover, the existence in the BD euthymic phase of alterations in the memory processes for the positive emotional stimuli might make patients more vulnerable to developing negative intrusive memories and depressive symptoms.

## Data Availability Statement

The datasets generated for this study are available on request to the corresponding author.

## Ethics Statement

All subjects gave written informed consent in accordance with the Declaration of Helsinki. The protocol was approved by the Department of Psychological, Health and Territorial Sciences’ ethical committee.

## Author Contributions

MA and BF conceived and designed the experiments. LS, AE, EA, and FP performed the experiments. CA, BF, and CP analyzed data. MA and BF wrote the article. AB and NM discussed the results and provided comments.

## Conflict of Interest Statement

The authors declare that the research was conducted in the absence of any commercial or financial relationships that could be construed as a potential conflict of interest.
